# *N*′-{(*E*)-[5-(Hy­droxy­meth­yl)furan-2-yl]methyl­idene}pyridine-4-carbohydrazide dihydrate

**DOI:** 10.1107/S1600536813020114

**Published:** 2013-07-27

**Authors:** M. K. Prasanna, M. Sithambaresan, K. Pradeepkumar, M. R. Prathapachandra Kurup

**Affiliations:** a Department of Chemistry and Research Centre, PRNSS College, Mattanur 670 702, Kannur, Kerala, India; b Department of Chemistry, Faculty of Science, Eastern University, Sri Lanka, Chenkalady, Sri Lanka; c Department of Applied Chemistry, Cochin University of Science and Technology, Kochi 682 022, India

## Abstract

In the title compound, C_12_H_11_N_3_O_3_·2H_2_O, the dihedral angle formed by the planes of the pyridine and the furan rings of the organic carbohydrazide mol­ecule is 4.66 (7)°. In the crystal, these mol­ecules form stacks along the *b*-axis direction, neighbouring mol­ecules within each stack being related by inversion and the shortest distance between the centroids of the pyridine and furan rings being 3.714 (1) Å. Mol­ecules from neighboring stacks are linked by pairs of N—H⋯O hydrogen bonds. The water mol­ecules fill the channels between the stacks being linked by O—H⋯O hydrogen bonds into helices along [010]. Besides this, water mol­ecules are involved in O—H⋯N and O—H⋯O hydrogen bonds with the carbohydrazide mol­ecules, thus forming a three-dimensional network, augmented by weak C—H⋯O interactions.

## Related literature

For biological properties of carbohydrazide and its derivatives, see: Rollas & Kucukguzel (2007 ▶); Bakir & Brown (2002 ▶). For the synthesis of related compounds, see: Sreeja & Kurup (2005 ▶). For related structures, see: Nair *et al.* (2012 ▶); Reshma *et al.* (2012 ▶); Prasanna *et al.* (2013 ▶).
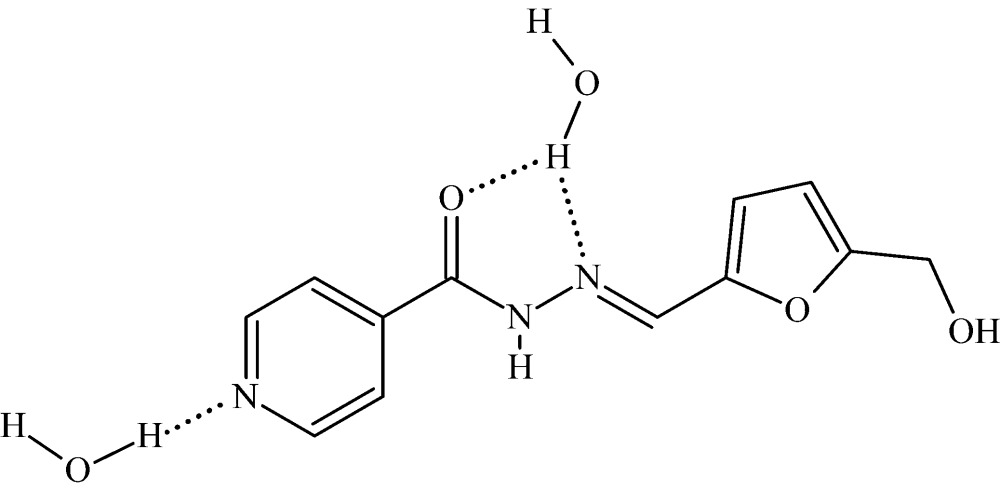


## Experimental

### 

#### Crystal data

C_12_H_11_N_3_O_3_·2H_2_O
*M* *_r_* = 281.27
Monoclinic, 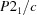

*a* = 10.7020 (14) Å
*b* = 7.0263 (8) Å
*c* = 18.024 (3) Å
β = 106.252 (7)°
*V* = 1301.2 (3) Å^3^
*Z* = 4
Mo *K*α radiation
μ = 0.11 mm^−1^
*T* = 296 K
0.50 × 0.25 × 0.25 mm

#### Data collection

Bruker Kappa APEXII CCD diffractometer
Absorption correction: multi-scan (*SADABS*; Bruker, 2007 ▶) *T*_min_ = 0.946, *T*_max_ = 0.972
9681 measured reflections
3139 independent reflections
2308 reflections with *I* > 2σ(*I*)
*R*_int_ = 0.023

#### Refinement

*R*[*F* ^2^ > 2σ(*F* ^2^)] = 0.038
*wR*(*F* ^2^) = 0.114
*S* = 1.03
3139 reflections
206 parameters
6 restraints
H atoms treated by a mixture of independent and constrained refinement
Δρ_max_ = 0.23 e Å^−3^
Δρ_min_ = −0.19 e Å^−3^

### 

Data collection: *APEX2* (Bruker, 2007 ▶); cell refinement: *SAINT* (Bruker, 2007 ▶); data reduction: *SAINT*; program(s) used to solve structure: *SHELXS97* (Sheldrick, 2008 ▶); program(s) used to refine structure: *SHELXL97* (Sheldrick, 2008 ▶); molecular graphics: *ORTEP-3 for Windows* (Farrugia, 2012 ▶) and *DIAMOND* (Brandenburg, 2010 ▶); software used to prepare material for publication: *SHELXL97* and *publCIF* (Westrip, 2010 ▶).

## Supplementary Material

Crystal structure: contains datablock(s) I, global. DOI: 10.1107/S1600536813020114/yk2097sup1.cif

Structure factors: contains datablock(s) I. DOI: 10.1107/S1600536813020114/yk2097Isup2.hkl

Supplementary material file. DOI: 10.1107/S1600536813020114/yk2097Isup3.cml

Additional supplementary materials:  crystallographic information; 3D view; checkCIF report

## Figures and Tables

**Table 1:** Hydrogen-bond geometry (Å, °)

*D*—H⋯*A*	*D*—H	H⋯*A*	*D*⋯*A*	*D*—H⋯*A*
O2*S*—H2*A*⋯N3	0.85 (2)	2.49 (2)	3.2272 (17)	146 (2)
O2*S*—H2*A*⋯O1	0.85 (2)	2.22 (2)	2.9648 (15)	146 (2)
O3—H3′⋯O1*S* ^i^	0.89 (2)	1.90 (2)	2.7937 (16)	174.9 (18)
O2*S*—H2*B*⋯O1*S* ^ii^	0.86 (2)	2.06 (2)	2.9145 (18)	171 (2)
O1*S*—H1*B*⋯O2*S* ^iii^	0.87 (2)	1.94 (2)	2.7924 (17)	167 (2)
N2—H2′⋯O3^iv^	0.876 (18)	2.024 (18)	2.8485 (16)	156.3 (16)
O1*S*—H1*A*⋯N1	0.87 (2)	2.03 (2)	2.8501 (16)	157 (2)
C4—H4⋯O3^iv^	0.93	2.50	3.3897 (18)	160
C12—H12*B*⋯O1^v^	0.97	2.43	3.3621 (19)	162

Symmetry codes: (i) 
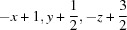
; (ii) 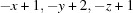; (iii) 
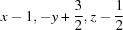
; (iv) 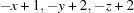; (v) 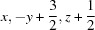.

**Fig. 1.**
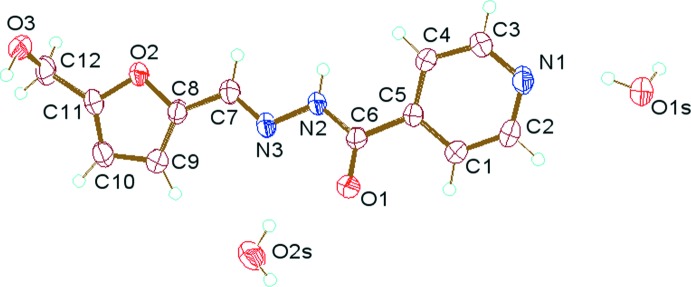
View of the structure of the title compound, showing the atom-labelling scheme and displacement ellipsoids for the non-H atoms drawn at the 50% probability level.

**Fig. 2.**
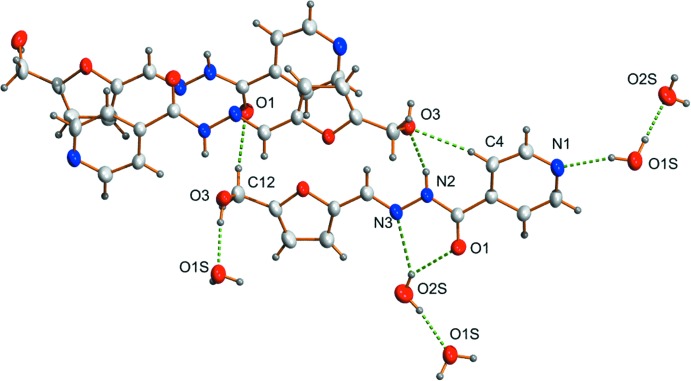
Hydrogen bonding in the crystal of the title compound.

**Fig. 3.**
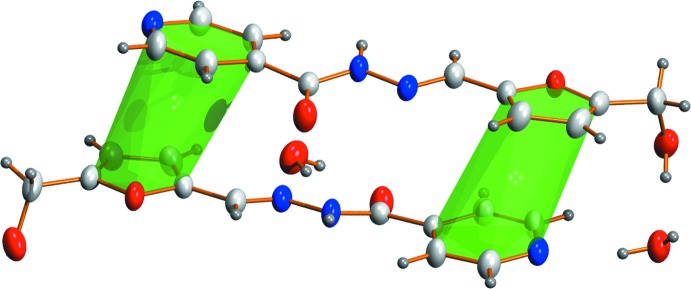
The π···π stacking interactions in the title compound.

**Fig. 4.**
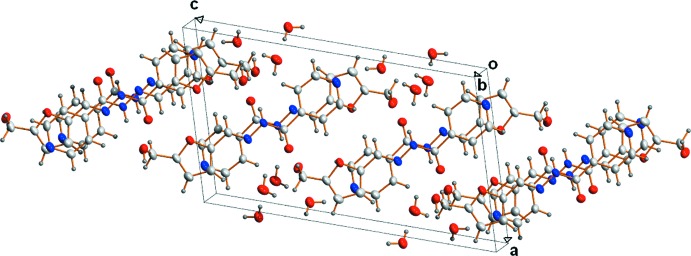
A view of the unit cell along the b axis.

## supplementary crystallographic information

### Comment

Heterocyclic carbohydrazides are compounds with a wide spectrum of biological and analytical applications. They form stable metal chelates which find applications in molecular sensing (Bakir & Brown, 2002). The title compound is a derivative of isoniazid which is one of the first line drug used in the treatment of tuberculosis. A number of hydrazones derived from isoniazid were reported to be active antitubercular agents and were found to be less toxic than isoniazid (Rollas & Kucukguzel, 2007).

The compound crystallizes in monoclinic *P*2_1_/*c* space group. The molecule exists in a *E* configuration with respect to the C7=N3 bond, typical of such kind of compounds (Reshma *et al.*, 2012), with the C8—C7—N3—N2 torsion angle of 178.04 (11)° (Fig 1). The N3—N2—C6—O1 torsion angle of -2.72 (18)° indicates the *cis* configuration of the O1 atom with respect to the hydrazine nitrogen atom N3 (Nair *et al.*, 2012). C7═N3 [1.267 (3) Å] and C6═O1 [1.217 (8) Å] bond distances are very close to the formal double C═N and C═O bond lengths (Prasanna, *et al.*, 2013) confirming that the carbohydrazide exists in solid state as an amido tautomer.

Seven classical hydrogen bonds are present in the crystal (Fig. 2). One of the nitrogen atoms of pyridine-4-carbohydrazide molecule is involved in classical intermolecular hydrogen bond of the N—H···O type with oxygen atom of the hydroxymethyl group of the neighboring molecule with a D···A distance of 2.8483 (16) Å. There are two hydrogen bonds of the O—H···O type between the two water molecules: O2S—H2B···O1S and O1S—H1B···O2S with D···A distances of 2.9146 (18) Å and 2.7923 (17) Å, respectively. Each water molecule is involved in other O—H···O and O—H···N hydrogen bonds: O2S—H2A···O1 and O3—H3'···O1S with D···A distances of 2.9647 (15) Å and 2.7937 (16) Å and O2S—H2A···N3 and O1S—H1A···N1 with D···A distances of 3.2272 (17) Å and 2.8501 (16) Å, respectively. Additionally, there are non-classical intermolecular C—H···O hydrogen bonds between the hydrogen atoms attached to C4 and C12 carbon atoms and the O3 and O1 atoms of the neighboring molecule with D···A distances of 3.390 (2) Å and 3.362 (2) Å, respectively. These interactions are augmented by weak π···π interactions which connect the molecules with a centroid-centroid distances of 3.714 (1) Å and 3.760 (1) Å (Fig. 3). The packing diagram showing the molecular assembly of the title compound along the *b* axis is shown in Fig. 4.

### Experimental

The title compound was prepared by adapting a reported procedure (Sreeja & Kurup, 2005). To a warm methanolic solution of 5-(hydroxymethyl)furan-2-carbaldehyde (0.126 g, 1 mmol), a methanolic solution of pyridine-4-carbohydrazide (0.137 g, 1 mmol) was added and the resulting solution was stirred well with slight heating for 75 minutes. A colorless compound formed was filtered off, washed with water, ethanol and finally with ether and dried. Single crystals suitable for X-ray diffraction studies were obtained by recrystallization from a mixture of methanol, ethanol and DMF.

### Refinement

All H atoms on C were placed in calculated positions, guided by difference maps, with C–H bond distances 0.93–0.97 Å. H atoms were assigned as *U*_iso_(H)=1.2Ueq(carrier) or 1.5Ueq (methyl C). The H atoms of the water molecule were located from difference maps and restrained using DFIX and DANG instructions. Omitted owing to disagreement was the reflection (1 1 0).

### Crystal data

**Table:**

C_12_H_11_N_3_O_3_·2H_2_O	*F*(000) = 592
*M**_r_* = 281.27	*D*_x_ = 1.436 Mg m^−^^3^
Monoclinic, *P*2_1_/*c*	Mo *K*α radiation, λ = 0.71073 Å
Hall symbol: -P 2ybc	Cell parameters from 3182 reflections
*a* = 10.7020 (14) Å	θ = 2.4–28.2°
*b* = 7.0263 (8) Å	µ = 0.11 mm^−^^1^
*c* = 18.024 (3) Å	*T* = 296 K
β = 106.252 (7)°	Block, colorless
*V* = 1301.2 (3) Å^3^	0.50 × 0.25 × 0.25 mm
*Z* = 4	

### Data collection

**Table:**

Bruker Kappa APEXII CCD diffractometer	3139 independent reflections
Radiation source: fine-focus sealed tube	2308 reflections with *I* > 2σ(*I*)
Graphite monochromator	*R*_int_ = 0.023
Detector resolution: 8.33 pixels mm^-1^	θ_max_ = 28.0°, θ_min_ = 3.1°
ω and φ scan	*h* = −14→14
Absorption correction: multi-scan (*SADABS*; Bruker, 2007)	*k* = −9→9
*T*_min_ = 0.946, *T*_max_ = 0.972	*l* = −23→16
9681 measured reflections	

### Refinement

**Table:**

Refinement on *F*^2^	Secondary atom site location: difference Fourier map
Least-squares matrix: full	Hydrogen site location: inferred from neighbouring sites
*R*[*F*^2^ > 2σ(*F*^2^)] = 0.038	H atoms treated by a mixture of independent and constrained refinement
*wR*(*F*^2^) = 0.114	*w* = 1/[σ^2^(*F*_o_^2^) + (0.0564*P*)^2^ + 0.1806*P*] where *P* = (*F*_o_^2^ + 2*F*_c_^2^)/3
*S* = 1.03	(Δ/σ)_max_ = 0.001
3139 reflections	Δρ_max_ = 0.23 e Å^−^^3^
206 parameters	Δρ_min_ = −0.19 e Å^−^^3^
6 restraints	Extinction correction: *SHELXL97* (Sheldrick, 2008), Fc^*^=kFc[1+0.001xFc^2^λ^3^/sin(2θ)]^-1/4^
Primary atom site location: structure-invariant direct methods	Extinction coefficient: 0.0056 (14)

### Special details

**Table:**

Geometry. All s.u.'s (except the s.u. in the dihedral angle between two l.s. planes) are estimated using the full covariance matrix. The cell s.u.'s are taken into account individually in the estimation of s.u.'s in distances, angles and torsion angles; correlations between s.u.'s in cell parameters are only used when they are defined by crystal symmetry. An approximate (isotropic) treatment of cell s.u.'s is used for estimating s.u.'s involving l.s. planes.
Refinement. Refinement of *F*^2^ against ALL reflections. The weighted *R*-factor *wR* and goodness of fit *S* are based on *F*^2^, conventional *R*-factors *R* are based on *F*, with *F* set to zero for negative *F*^2^. The threshold expression of *F*^2^ > σ(*F*^2^) is used only for calculating *R*-factors(gt) *etc*. and is not relevant to the choice of reflections for refinement. *R*-factors based on *F*^2^ are statistically about twice as large as those based on *F*, and *R*- factors based on ALL data will be even larger.

### Fractional atomic coordinates and isotropic or equivalent isotropic displacement parameters (Å^2^)

**Table:**

	*x*	*y*	*z*	*U*_iso_*/*U*_eq_	
O2	0.65878 (8)	0.90209 (13)	1.02711 (5)	0.0361 (2)	
N3	0.59249 (10)	0.91380 (15)	0.82424 (6)	0.0352 (3)	
N1	0.17508 (11)	0.89401 (16)	0.49077 (6)	0.0405 (3)	
N2	0.48334 (11)	0.90481 (16)	0.76219 (6)	0.0340 (3)	
O3	0.74289 (10)	1.09936 (16)	1.18406 (6)	0.0456 (3)	
O1	0.60410 (9)	0.97315 (16)	0.68198 (6)	0.0459 (3)	
O1S	0.05219 (11)	0.82845 (18)	0.33090 (6)	0.0484 (3)	
O2S	0.87042 (11)	0.96561 (18)	0.78948 (7)	0.0553 (3)	
C1	0.40194 (13)	0.8935 (2)	0.55313 (8)	0.0379 (3)	
H1	0.4862	0.8853	0.5486	0.046*	
C5	0.38137 (12)	0.91494 (16)	0.62455 (7)	0.0291 (3)	
C4	0.25460 (13)	0.9228 (2)	0.62766 (8)	0.0382 (3)	
H4	0.2358	0.9350	0.6748	0.046*	
C2	0.29770 (15)	0.8844 (2)	0.48870 (8)	0.0427 (3)	
H2	0.3138	0.8708	0.4409	0.051*	
C3	0.15595 (13)	0.9122 (2)	0.55978 (8)	0.0421 (3)	
H3	0.0706	0.9181	0.5626	0.051*	
C7	0.57428 (13)	0.89688 (18)	0.89043 (8)	0.0353 (3)	
H7	0.4906	0.8833	0.8955	0.042*	
C8	0.68443 (13)	0.89898 (17)	0.95717 (7)	0.0328 (3)	
C11	0.77674 (13)	0.90344 (18)	1.08159 (7)	0.0348 (3)	
C10	0.87211 (14)	0.8998 (2)	1.04728 (9)	0.0439 (4)	
H10	0.9610	0.8996	1.0719	0.053*	
C12	0.77600 (15)	0.9152 (2)	1.16286 (8)	0.0441 (4)	
H12B	0.7141	0.8236	1.1719	0.053*	
H12A	0.8615	0.8810	1.1956	0.053*	
C9	0.81354 (14)	0.8965 (2)	0.96715 (9)	0.0437 (3)	
H9	0.8559	0.8931	0.9286	0.052*	
C6	0.49926 (12)	0.93343 (17)	0.69202 (7)	0.0304 (3)	
H1A	0.067 (2)	0.853 (3)	0.3797 (9)	0.089 (7)*	
H2'	0.4086 (18)	0.875 (2)	0.7700 (10)	0.058 (5)*	
H1B	−0.0138 (16)	0.751 (3)	0.3174 (11)	0.077 (6)*	
H2B	0.888 (2)	1.017 (3)	0.7503 (11)	0.094 (8)*	
H3'	0.808 (2)	1.176 (3)	1.1820 (11)	0.075 (6)*	
H2A	0.7875 (15)	0.961 (3)	0.7772 (12)	0.083 (7)*	

### Atomic displacement parameters (Å^2^)

**Table:**

	*U*^11^	*U*^22^	*U*^33^	*U*^12^	*U*^13^	*U*^23^
O2	0.0314 (5)	0.0508 (5)	0.0229 (5)	−0.0029 (4)	0.0023 (4)	0.0017 (4)
N3	0.0323 (6)	0.0432 (6)	0.0257 (6)	−0.0023 (4)	0.0009 (5)	−0.0009 (4)
N1	0.0406 (7)	0.0478 (7)	0.0282 (6)	−0.0018 (5)	0.0016 (5)	−0.0017 (5)
N2	0.0276 (6)	0.0488 (6)	0.0224 (6)	−0.0037 (5)	0.0019 (5)	0.0008 (4)
O3	0.0446 (6)	0.0623 (7)	0.0327 (6)	−0.0122 (5)	0.0156 (5)	−0.0072 (4)
O1	0.0300 (5)	0.0750 (7)	0.0327 (5)	−0.0025 (5)	0.0089 (4)	0.0011 (5)
O1S	0.0448 (6)	0.0656 (7)	0.0315 (6)	−0.0005 (5)	0.0052 (5)	−0.0008 (5)
O2S	0.0374 (6)	0.0725 (8)	0.0497 (7)	0.0006 (5)	0.0019 (5)	0.0059 (6)
C1	0.0339 (7)	0.0518 (8)	0.0293 (7)	0.0009 (6)	0.0107 (6)	−0.0013 (5)
C5	0.0307 (6)	0.0311 (6)	0.0241 (6)	0.0005 (5)	0.0053 (5)	0.0013 (4)
C4	0.0344 (7)	0.0559 (8)	0.0247 (7)	0.0008 (6)	0.0088 (5)	0.0010 (5)
C2	0.0474 (8)	0.0551 (8)	0.0246 (7)	−0.0006 (6)	0.0086 (6)	−0.0048 (6)
C3	0.0317 (7)	0.0582 (9)	0.0334 (8)	−0.0005 (6)	0.0042 (6)	0.0006 (6)
C7	0.0333 (7)	0.0427 (7)	0.0271 (7)	−0.0013 (5)	0.0040 (5)	0.0007 (5)
C8	0.0363 (7)	0.0372 (6)	0.0233 (6)	−0.0017 (5)	0.0058 (5)	−0.0013 (5)
C11	0.0326 (7)	0.0378 (7)	0.0279 (7)	−0.0010 (5)	−0.0018 (5)	0.0012 (5)
C10	0.0327 (7)	0.0562 (9)	0.0377 (8)	0.0011 (6)	0.0010 (6)	−0.0092 (6)
C12	0.0474 (8)	0.0527 (8)	0.0255 (7)	−0.0084 (6)	−0.0006 (6)	0.0065 (6)
C9	0.0369 (7)	0.0594 (9)	0.0347 (8)	−0.0013 (6)	0.0100 (6)	−0.0095 (6)
C6	0.0289 (6)	0.0350 (6)	0.0264 (6)	0.0022 (5)	0.0065 (5)	−0.0005 (5)

### Geometric parameters (Å, º)

**Table:**

O2—C8	1.3634 (15)	C1—H1	0.9300
O2—C11	1.3654 (15)	C5—C4	1.3745 (18)
N3—C7	1.2671 (17)	C5—C6	1.4928 (17)
N3—N2	1.3741 (15)	C4—C3	1.3763 (18)
N1—C3	1.3221 (17)	C4—H4	0.9300
N1—C2	1.3248 (19)	C2—H2	0.9300
N2—C6	1.3379 (16)	C3—H3	0.9300
N2—H2'	0.876 (18)	C7—C8	1.4294 (18)
O3—C12	1.4218 (18)	C7—H7	0.9300
O3—H3'	0.89 (2)	C8—C9	1.3427 (19)
O1—C6	1.2178 (15)	C11—C10	1.333 (2)
O1S—H1A	0.865 (16)	C11—C12	1.4694 (19)
O1S—H1B	0.872 (15)	C10—C9	1.405 (2)
O2S—H2B	0.860 (16)	C10—H10	0.9300
O2S—H2A	0.853 (15)	C12—H12B	0.9700
C1—C2	1.3683 (19)	C12—H12A	0.9700
C1—C5	1.3737 (18)	C9—H9	0.9300
			
C8—O2—C11	106.27 (10)	N3—C7—C8	118.91 (12)
C7—N3—N2	116.25 (11)	N3—C7—H7	120.5
C3—N1—C2	116.53 (12)	C8—C7—H7	120.5
C6—N2—N3	117.28 (11)	C9—C8—O2	110.02 (11)
C6—N2—H2'	123.5 (12)	C9—C8—C7	133.47 (13)
N3—N2—H2'	119.1 (12)	O2—C8—C7	116.50 (11)
C12—O3—H3'	106.2 (12)	C10—C11—O2	109.88 (12)
H1A—O1S—H1B	108.4 (18)	C10—C11—C12	132.98 (13)
H2B—O2S—H2A	104.8 (19)	O2—C11—C12	117.09 (12)
C2—C1—C5	119.61 (12)	C11—C10—C9	107.34 (13)
C2—C1—H1	120.2	C11—C10—H10	126.3
C5—C1—H1	120.2	C9—C10—H10	126.3
C1—C5—C4	117.48 (12)	O3—C12—C11	112.99 (11)
C1—C5—C6	116.89 (11)	O3—C12—H12B	109.0
C4—C5—C6	125.60 (11)	C11—C12—H12B	109.0
C5—C4—C3	118.81 (12)	O3—C12—H12A	109.0
C5—C4—H4	120.6	C11—C12—H12A	109.0
C3—C4—H4	120.6	H12B—C12—H12A	107.8
N1—C2—C1	123.54 (13)	C8—C9—C10	106.49 (13)
N1—C2—H2	118.2	C8—C9—H9	126.8
C1—C2—H2	118.2	C10—C9—H9	126.8
N1—C3—C4	124.02 (13)	O1—C6—N2	122.77 (12)
N1—C3—H3	118.0	O1—C6—C5	120.18 (11)
C4—C3—H3	118.0	N2—C6—C5	117.05 (11)
			
C7—N3—N2—C6	176.19 (11)	C8—O2—C11—C12	177.41 (11)
C2—C1—C5—C4	1.17 (19)	O2—C11—C10—C9	0.14 (16)
C2—C1—C5—C6	−177.08 (12)	C12—C11—C10—C9	−177.28 (15)
C1—C5—C4—C3	−1.08 (19)	C10—C11—C12—O3	103.43 (18)
C6—C5—C4—C3	177.01 (12)	O2—C11—C12—O3	−73.84 (15)
C3—N1—C2—C1	−0.4 (2)	O2—C8—C9—C10	−0.54 (16)
C5—C1—C2—N1	−0.4 (2)	C7—C8—C9—C10	−179.70 (14)
C2—N1—C3—C4	0.5 (2)	C11—C10—C9—C8	0.25 (17)
C5—C4—C3—N1	0.2 (2)	N3—N2—C6—O1	−2.72 (18)
N2—N3—C7—C8	178.04 (11)	N3—N2—C6—C5	177.35 (10)
C11—O2—C8—C9	0.62 (14)	C1—C5—C6—O1	16.96 (17)
C11—O2—C8—C7	179.94 (11)	C4—C5—C6—O1	−161.14 (13)
N3—C7—C8—C9	−8.1 (2)	C1—C5—C6—N2	−163.11 (12)
N3—C7—C8—O2	172.79 (11)	C4—C5—C6—N2	18.79 (18)
C8—O2—C11—C10	−0.46 (14)		

### Hydrogen-bond geometry (Å, º)

**Table:**

*D*—H···*A*	*D*—H	H···*A*	*D*···*A*	*D*—H···*A*
O2*S*—H2*A*···N3	0.85 (2)	2.49 (2)	3.2272 (17)	146 (2)
O2*S*—H2*A*···O1	0.85 (2)	2.22 (2)	2.9648 (15)	146 (2)
O3—H3′···O1*S*^i^	0.89 (2)	1.90 (2)	2.7937 (16)	174.9 (18)
O2*S*—H2*B*···O1*S*^ii^	0.86 (2)	2.06 (2)	2.9145 (18)	171 (2)
O1*S*—H1*B*···O2*S*^iii^	0.87 (2)	1.94 (2)	2.7924 (17)	167 (2)
N2—H2′···O3^iv^	0.876 (18)	2.024 (18)	2.8485 (16)	156.3 (16)
O1*S*—H1*A*···N1	0.87 (2)	2.03 (2)	2.8501 (16)	157 (2)
C4—H4···O3^iv^	0.93	2.50	3.3897 (18)	160
C12—H12*B*···O1^v^	0.97	2.43	3.3621 (19)	162

Symmetry codes: (i) −*x*+1, *y*+1/2, −*z*+3/2; (ii) −*x*+1, −*y*+2, −*z*+1; (iii) *x*−1, −*y*+3/2, *z*−1/2; (iv) −*x*+1, −*y*+2, −*z*+2; (v) *x*, −*y*+3/2, *z*+1/2.
